# Acute effects of fresh versus dried Hayward green kiwifruit on sleep quality, mood, and sleep-related urinary metabolites in healthy young men with good and poor sleep quality

**DOI:** 10.3389/fnut.2023.1079609

**Published:** 2023-03-14

**Authors:** Alexander P. Kanon, Caroline Giezenaar, Nicole C. Roy, Warren C. McNabb, Sharon J. Henare

**Affiliations:** ^1^School of Health Sciences, College of Health, Massey University, Palmerston North, New Zealand; ^2^Riddet Institute, Massey University, Te Ohu Rangahau Kai Facility, Palmerston North, New Zealand; ^3^Alpha-Massey Natural Nutraceutical Research Centre, Massey University, Palmerston North, New Zealand; ^4^Food Experience and Sensory Testing Laboratory, School of Food and Advanced Technology, Massey University, Palmerston North, New Zealand; ^5^Department of Human Nutrition, University of Otago, Dunedin, New Zealand; ^6^High-Value Nutrition National Science Challenge, Auckland, New Zealand

**Keywords:** sleep, mood, kiwifruit, melatonin (6-sulfatoxymelatonin), serotonin, alertness

## Abstract

**Background and aims:**

Daily kiwifruit (KF) consumption has been associated with improved sleep quality, but underlying physiological mechanisms are unknown. This study examined acute effects of fresh and dried green KF, compared with a water control, on sleep quality, mood, and urinary serotonin and melatonin metabolite concentrations.

**Methods:**

24 men (age: 29 ± 1 years, body mass index: 24 ± 1 kg/m^2^) with poor (*n* = 12) or good (*n* = 12) sleep quality participated in a randomized, single-blind crossover study. One of three treatments was consumed with a standardized evening meal; (1) the flesh of two fresh green KF, (2) dried green KF powder (including skin; equivalent to dry matter of two fresh KF) mixed with water, or (3) a water control, in their own home. Subjective and objective sleep quality, mood, waking urinary 5-hydroxyindoleacetic acid (5-HIAA), 6-sulfatoxymelatonin (aMT6s), vitamin C and B-vitamin concentrations were determined.

**Results:**

Regardless of sleep quality group, compared to control, morning sleepiness, alertness upon awakening, and vigor were improved (*p* < 0.05) after dried KF consumption. Compared to control, both fresh and dried KF treatments tended (*p* < 0.1) toward improved esteem and total mood disturbance. Both KF treatments increased (fresh +1.56 ± 0.4 ng/g, *p* = 0.001; dried: +1.30 ± 0.4 ng/g, *p* = 0.004) urinary concentration of the serotonin metabolite 5-HIAA compared to the control (4.32 ± 0.4 ng/g). In poor sleepers, ease of awakening improved by 24% after dried KF consumption (*p* = 0.005) and tended to improve by 13% after fresh KF intake (*p* = 0.052) compared to the control. Good sleepers tended toward 9% improved ratings of getting to sleep with fresh KF (*p* = 0.053) compared to the control. Poor sleepers had lower amounts of some B-vitamins compared to good sleepers (*p* < 0.05).

**Conclusion:**

Consumption of dried or fresh KF with a standard evening meal, was associated with improved aspects of sleep quality and mood, possibly mediated through changes in serotonin metabolism.

**Clinical trial registration:**

[www.anzctr.org.au], identifier [ACTRN12621000046808].

**Graphical Abstract:**
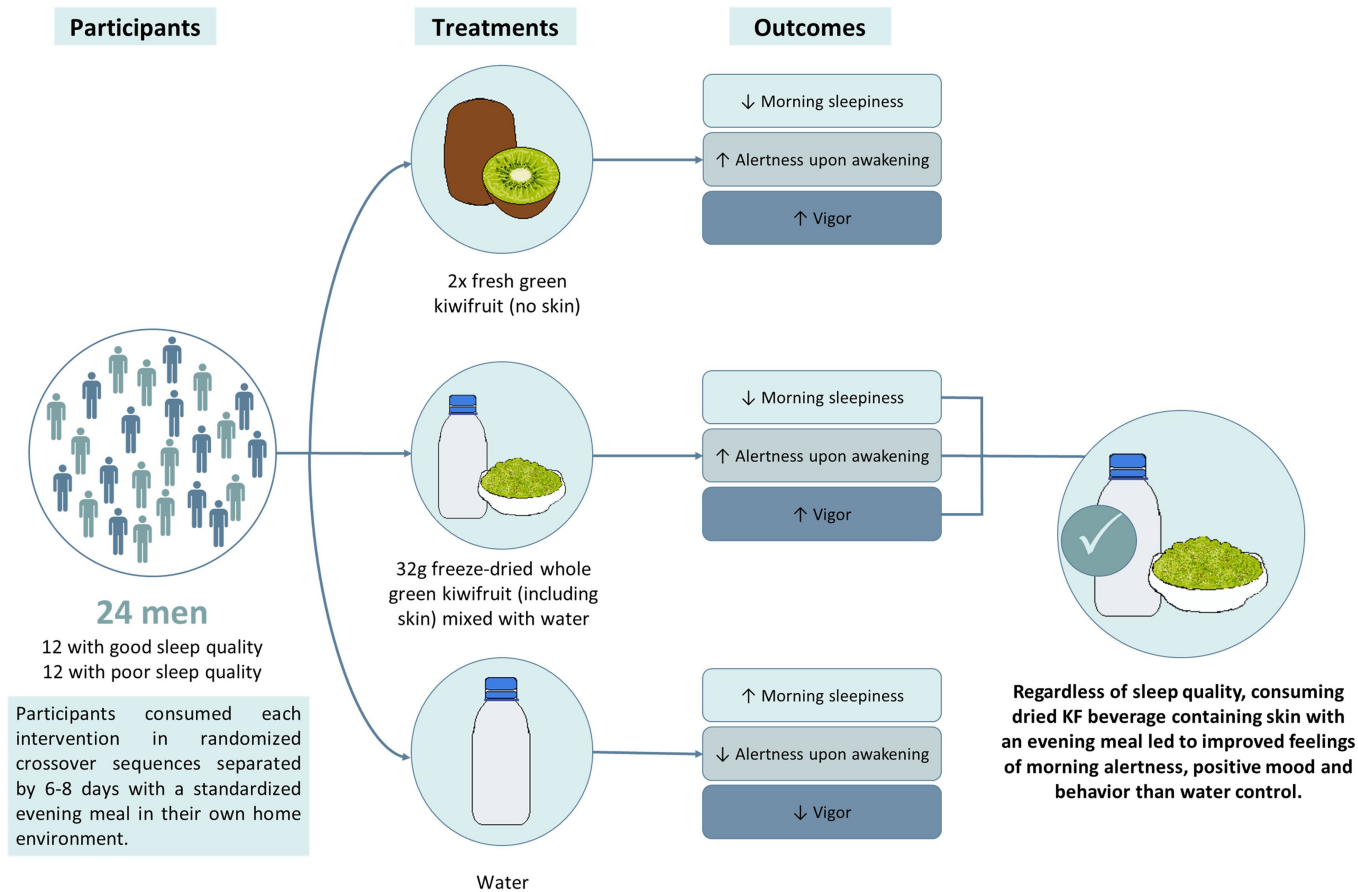

## 1. Introduction

Sleep is essential for the cellular repair of the body. Inadequate sleep is associated with increased health risks such as inflammation, diabetes, hypertension, and obesity. (1–5). Insufficient sleep can also lower cognitive performance and cause mood changes (6). One night of sleep disturbance can affect a person’s ability to concentrate; increasing technical errors and their overall mood the following day (7–9). Sleep quality is an assessment of a person’s contentment with their sleep period. This includes measures of one’s sleep duration, latency, efficiency, and waking after sleep. Good sleep quality has positive effects including improved daily function and feeling rested. Poor sleep quality outcomes include increased irritability and fatigue (10).

The circadian clock in the hypothalamus, which is affected by the light and dark cycles, controls sleep–wake cycles in humans. The neurohormone melatonin is secreted from pinealocytes in the pineal gland, and its precursor serotonin plays a vital role in regulating this. The urinary melatonin metabolite 6-sulfatoxymelatonin (aMT6s) is related to subjective and objective sleep quality measures (11) and the urinary concentration of the serotonin metabolite 5-hydroxyindoleacetic acid (5-HIAA) is related to mood (12). Moreover, first-morning urine concentration of aMT6s accurately reflected peak and total nocturnal plasma melatonin secretion (13).

The consumption of fruit and vegetables is known to affect plasma and urinary melatonin and serotonin metabolite concentrations. Consumption of vegetables (sweet corn, bitter gourd, Japanese radish sprout, *shumeji* and *shiitake* mushrooms) (14), tomatoes (15), and cherries (16), which are rich in melatonin, increased morning urinary aMT6s concentration (14–16) and improved sleep quality (15–17). Consumption of serotonin-rich fruits, such as kiwifruit (KF) (18) and a Jerte Valley cherry-based product (19) increased urinary levels of 5-HIAA (19, 20) and mood (19). Additionally, vitamin C is a co-factor involved in catecholamine biosynthesis and reducing oxidative stress. Studies have also shown increased urinary levels of vitamin C when consuming vitamin C-rich foods. B-vitamins are also important co-factors involved in catecholamine biosynthesis. Furthermore, daily consumption of two green KF 1 h before bed for 4 weeks was associated with improved sleep quality (21). KF is high in serotonin, vitamin C and B-vitamins (22). However, the relationships between biochemical measures and sleep quality with KF consumption are unknown.

The aim of this study was to determine the effect of consuming the flesh of two fresh green KF (without the skins), whole freeze-dried green KF (with the skins included; equivalent to two green KF), or a water control with a standardized evening meal on urinary concentrations of metabolites of melatonin, serotonin, vitamin C and B-vitamins as well as objective (actigraphy) and subjective sleep quality and mood measures in healthy young men. We hypothesized that sleep quality and mood would improve and urinary metabolite concentrations would increase after the KF interventions compared to the control intervention.

## 2. Materials and methods

### 2.1. Participant recruitment and screening

Twenty-four healthy men (eligibility: age 18–35 years, Body Mass Index (BMI) 18.5–30 kg/m^2^) were recruited by advertisement from the Massey University campus and community in Palmerston North, Manawatu, New Zealand, between January 2021 and May 2021 amid the COVID-19 pandemic. The Pittsburgh Sleep Quality Index (PSQI) was used to determine participants subjective sleep quality. Participants were classified as a ‘good sleeper’ when their global PSQI score was ≤ 5 and as a ‘poor sleeper’ when their global PSQI score was > 5 (23). Exclusion criteria included smoking, excessive alcohol intake of > 21 standard drinks per week, use of prescribed or non-prescribed medications and antibiotics, physically active for more than 2 hours a day, food intolerances and allergies, consuming a vegan/vegetarian diet, gastrointestinal disorders; chronic conditions, such as cardiorespiratory, diabetes mellitus, high cholesterol/blood pressure; psychiatric conditions; diagnosed sleep conditions and working night shift or irregular work hours. In addition, participants who experienced significant weight loss (>5%) 3 months prior to the start of the study or consumed strict diets were excluded.

The study protocol was approved by the Massey University Human Ethics Committee (Massey University HEC: Southern A application-20/52), and the study was conducted according to the guidelines in the Declaration of Helsinki. The study was registered as a clinical trial with the Australian New Zealand Clinical Trial Registry.^1^ All participants provided written informed consent before the clinical trial and could withdraw at any time for any reason.

### 2.2. Study design, intervention, and protocol

The intervention was a randomized, single-blind, cross-over study. The study involved two green KF interventions of different forms and a control. The two KF interventions were: (1) flesh of two fresh green KF (*Actinidia deliciosa* cv. Hayward) (flesh only; approximate 200 g), (2) freeze-dried whole (flesh and skin), green KF (32 g, equivalent to the dry matter of two fresh green KF) with 200 ml of water, or (3) a control of 200 ml of water. The nutritional composition of each treatment is shown in Table 1. The researcher administering and analyzing the data (AK) was blinded to treatment allocation until completion of analyzes, and a separate researcher (CG) was unblinded and responsible for preparing the interventions. The researcher preparing the interventions was not involved in analyzing the data.

**Table 1:** Nutritional composition of total daily treatments for the fresh kiwifruit, freeze-dried kiwifruit, and control (water).

Nutrient	Quantity per serve		
	≈200 g fresh kiwifruit^a^	32 g freeze-dried whole green kiwifruit powder with 200 ml water^b^	200 ml water
*Energy* (kcal)	95.00	96.00	0
*Carbohydrates*			
Carbohydrate, total (g)	18.20	21.30	0
Sugars			
Sugar, total (g)	17.60	20.77	0
Fructose (g)	9.40	10.30	0
Galactose (g)	0.00	0.03	0
Glucose (g)	8.20	8.93	0
Lactose Anhydrous (g)	0.00	0.03	0
Lactose monohydrate (g)	0.00	0.03	0
Maltose (g)	0.00	0.03	0
Sucrose (g)	0.00	1.41	0
Dietary fiber, total (g)	6.00	4.16	0
*Fats*			
Fat, total (g)	1.40	0.61	0
*Minerals*			
Calcium (mg)	54.00	41.92	0
Iron (mg)	0.44	0.26	0
Potassium (mg)	602.00	550.40	0
Magnesium (mg)	28.00	18.24	0
Sodium (mg)	4.00	6.05	0
Zinc (mg)	0.20	0.16	0
*Protein and amino acid*			
Protein (g)	2.40	1.31	0
Tryptophan (mg)	100.00	43.10	0
*Vitamins Water Soluble*			
Vitamin B_12_	0.00	0.02	0
Vitamin B_3_ (mg)	1.66	0.40	0
Vitamin B_6_ (mg)	0.14	0.36	0
Vitamin B_9_ (mg)	76.00	48.00	0
Vitamin C (mg)	170.20	98.56	0
*Vitamins Fat Soluble*			
Vitamin E (mg)	1.72	2.69	0
*Other*			
Ash (g)	1.40	1.18	0
Water (g)	167.00	200.00	200.00
Total phenolic content (mg GAE)^b^	168.30**	217.60**	0

a Determined on the flesh of ripe ready-to-eat fruit, with skin removed. Data from the Concise New Zealand Food Composition tables (24).

b Determined in the whole dried green KF powder.

Analysis conducted by the Nutrition Laboratory, Massey University, Palmerston North. GAE, Gallic Acid Equivalence; ^**^p < 0.01 represents the statistical difference between fresh and dried KF.

**Figure 1:**
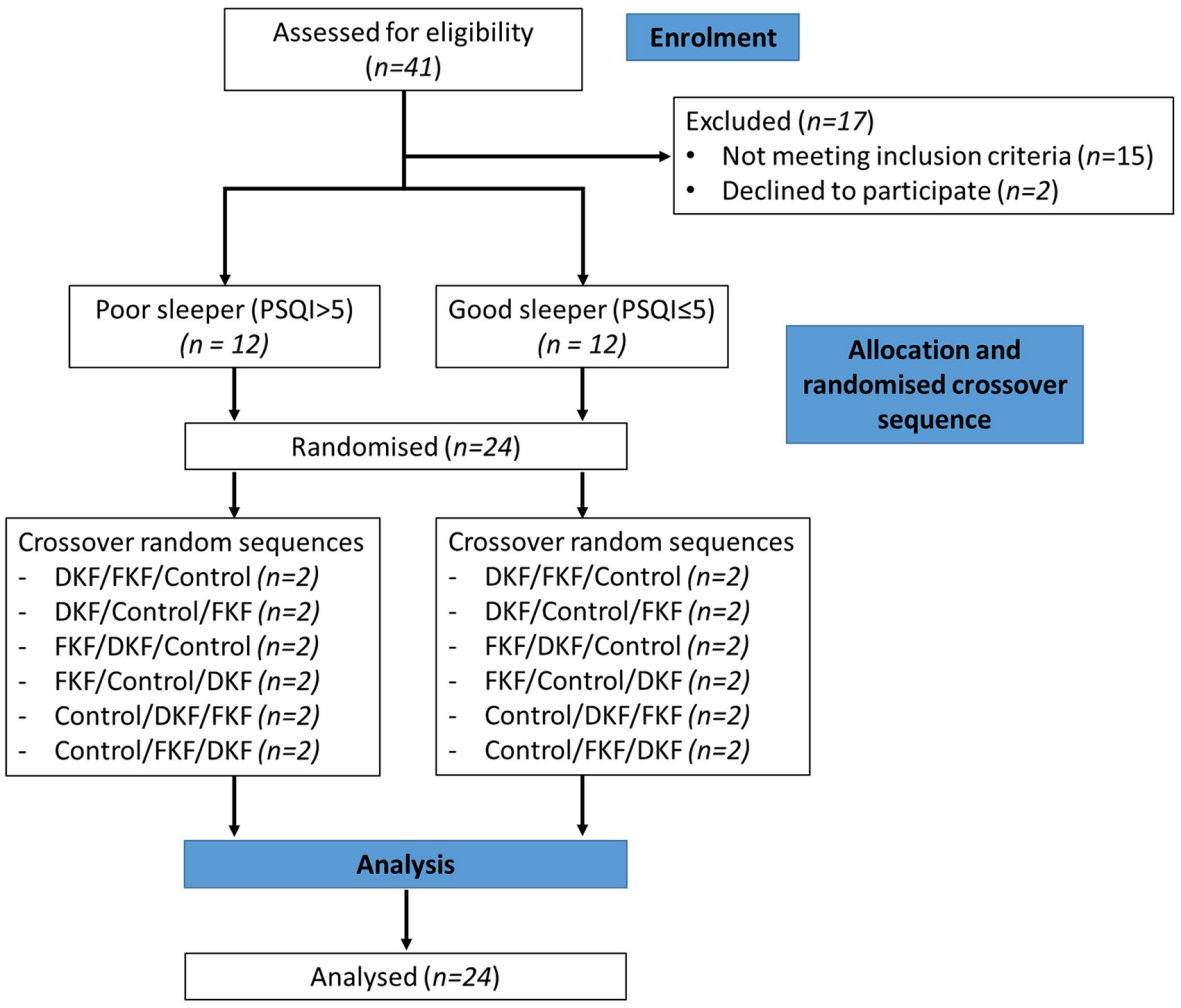
Consolidated Standards of Reporting Trials (CONSORT) flow diagram of the recruitment, enrolment, and random assignment process. DKF (Dried KF—32 g freeze-dried green KF powder), FKF (fresh KF—approx. 200 g fresh green KF).

Randomization was conducted using a 6 × 3 Williams Design balanced for the order of presentation and carryover effects. The trial CONSORT flow diagram is shown in Figure 1. The study was conducted in the participant’s home environment. Before starting the study evenings, a familiarization evening occurred one to three days before the first evening. The same procedure described below was followed without providing a standardized evening meal and intervention.

**Figure 2:**
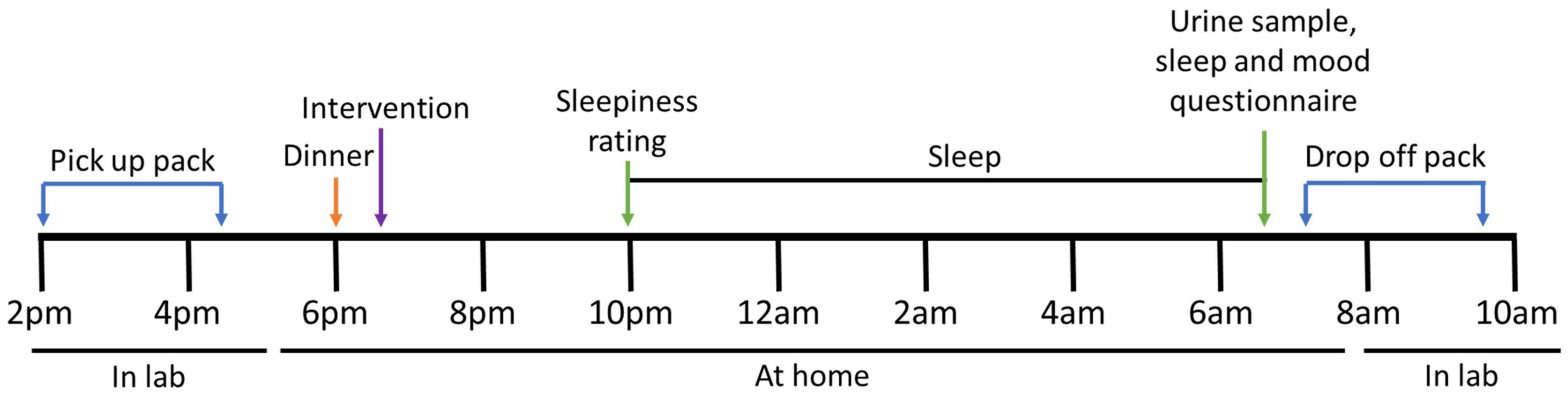
Study protocol for each intervention. At approximately 6 pm, the participant consumed a standardized evening dinner followed by one of three interventions. Before bedtime, at approximately 10–11 pm, the participant was asked to rate their sleepiness. Upon waking the next morning, a urine sample was collected, and the survey booklet was completed. The sample was delivered to the laboratory within 2 h of collection.

Participants were studied on three separate occasions, separated by six to eight days. Each participant consumed an intervention with a standardized evening meal [Pub Size Spaghetti and Meatballs (McCain Foods), ∼720 kcal, P 26.5 g, C 75.0 g, F 33.5 g] Enrolled participants were asked to refrain from eating KF from enrolment until the completion of the study period. On the day of the study, participants were asked to collect a data collection pack (Figure 2). The pack contained the evening meal, an intervention they were randomized to receive, an actigraphy watch, a urine sample collection container, and a survey booklet. It was confirmed that the participant refrained from consuming any restricted foods during the two days prior to their study day. The restricted foods included oranges, pineapples, bananas, mangos, papayas, plums, grape, cherries, strawberries, tomatoes, capsicum, pistachios, plantains, mushrooms, chocolate, teas, coffee, and caffeinated beverages as they are known to contain and alter levels of serotonin and melatonin in urine (20, 25, 26).

The participant consumed the standardized evening meal 4 h before their usual sleep time, followed immediately by the allocated intervention taking note of the time. Participants were asked not to eat any food or drinks, except water, until the following morning. Immediately before going to bed, participants were asked to rate their sleepiness level using the Stanford Sleepiness Scale (SSS). The participant was asked to activate the phase marker on the actigraphy watch when they were in bed and ready to sleep. Upon waking the following day, participants were asked to activate the phase marker, collect the whole first-morning urine sample noting the time, and complete a set of surveys including another SSS, Leeds Sleep Evaluation Questionnaire (LSEQ), and the abbreviated Profile of Mood States (POMS). The urine sample and surveys were delivered to the laboratory within 2 h of waking (Figure 2). Arrangements were made if the participant could not deliver samples within this time.

#### 2.2.1. Subjective measures for sleep quality and mood

Two questionnaires to measure subjective sleep quality were used. The SSS measures sleepiness, consisting of a one-item scale; the participants select one of seven statements that best represent their current sleepiness. For example, a score of one indicates feeling active, vital, and alert, while a score of seven indicates no longer fighting sleep, sleep onset soon (27). The LSEQ is ten Visual Analog Scales (VAS) as a subjective self-measure to assess sleep quality changes throughout psychopharmacological treatment interventions (28). The scale evaluates four domains of sleep: (i) ease of getting to sleep (mean of questions 1, 2 and 3), (ii) quality of sleep (mean of questions 4 and 5), (iii) ease of awakening from sleep (mean of questions 6 and 7), and (iv) alertness upon awakening (mean of questions 8, 9, and 10). A 100 mm VAS scale was used for scoring, and scores were averaged per domain to determine the domain score. Higher scores indicate a better sleep quality domain.

Mood was assessed using the abbreviated POMS questionnaire (29). This form contains 40 mood-related adjectives rated on a 5-point Likert-type scale, ranging from 0 (not at all) to 4 (extremely). The data is then categorized into seven mood scales (maximum scores indicated): (i) tension, (ii) depression, (iii) anger, (iv) fatigue, (v) confusion, (vi) vigor, and (vii) esteem. Finally, a Total Mood Score (TMD) was calculated by adding tension, depression, anger, fatigue, and confusion scores, then subtracting the vigor and esteem scores.

#### 2.2.2. Objective sleep measures

Participants were provided with an Actiwatch Spectrum Plus (Philips Respironics, Murrysville, Pennsylvania, United States) to wear on the wrist of their non-dominant arm during the study evenings. The medium threshold setting was used due to its use in studies using actigraphy to assess sleep (30), and 30 s was selected as the epoch length. The data was downloaded and subject to a mathematical algorithm in Actiware 6.0.9 (Philips Respironics, Murrysville, Pennsylvania, USA) to quantify sleep onset latency, sleep efficiency, total sleep time, wake after sleep onset, number of awakenings and the average length of awakenings.

#### 2.2.3. Biochemical measures

Urine samples were collected in 500 ml containers with 1.0 g of ethylenediaminetetraacetic acid (EDTA). Participants were provided with two containers. Upon arriving at the laboratory, urine samples were weighed, and their volume was recorded. The urine was aliquoted into 1.5 ml Eppendorf tubes and stored at -80°C until analysis of aMT6s and vitamin C. Ten milliliters of urine were acidified with 6 M HCL and aliquoted and stored at -80°C until analysis of 5-HIAA. Urinary aMT6s and 5-HIAA analyzes were analyzed according to the manufacturer’s instructions using an enzyme-linked immunosorbent assay (ELISA) kit (IBL International, Hamburg, Germany). Assays were performed in duplicate and averaged. The average intra-assay coefficient of variance for the aMT6s assay was 5.2%, and the inter-assay was 2.9%. The average intra-assay coefficient of variance for the 5-HIAA assay was 7.5%, and the inter-assay was 3.5%. The ascorbate content of the samples was determined by reverse-phase high-performance liquid chromatography (HPLC) with electrochemical detection (31). B-vitamins and vitamers were measured using high-performance liquid chromatography coupled with mass spectrometry (UHPLC–MS/MS) technique (32). The B-vitamins measured included pantothenic acid, 4-pyridoxic acid, nicotinic acid, nicotinamide, nicotinuric acid, pyridoxal, biotin, riboflavin, folic acid, pyridoxamine and thiamine. The concentrations of measured metabolites and vitamins were corrected to creatinine to adjust for variation in urine dilution. Creatinine was measured using the colorimetric Jaffe method (33) (Nutrition Laboratory, Massey University).

### 2.3. Data and statistical analysis

A power analysis was performed using ease of getting to sleep VAS from LSEQ as the dependent variable to estimate the required sample size. Estimates of variance components were conducted based on data from a study examining caffeine ingestion’s effect on VAS getting to sleep in healthy subjects (34). When there are three treatments and a sample size of two in each of the six sequences, a 6 × 3 Williams Crossover inequality test of paired differences will have 94.2% power to detect a minimum difference of 20 mm or greater, assuming that the standard deviation of the paired differences is 13 mm (34) at the 5% significance level. The Bonferroni adjustment was used to keep the family-wise error at the specified error level. Thus, with the total number of pairwise comparisons equal to 3, each pairwise test was at the two-sided 1.67% significance level.

Statistical analyzes were performed using SPSS software (version 25; IBM, Armonk, NY, United States). No outliers for any outcome measures were noted by examination of studentized residuals for values greater than ±3. A paired *t*-test was used to compare nutritional compositional and demographic data. Effects of sleeper type and treatment and their interaction effect were determined using a repeated measures mixed-effects model. An unstructured covariance structure was used to account for the repeated treatments by the subject. *Post hoc* comparisons, adjusted for multiple comparisons using Bonferroni’s correction, were performed when there were significant main or interaction effects. Residual plots were inspected to confirm that the normality and constant variance model assumptions were met. Statistical significance of the mixed effects models was accepted at a probability inferior to 0.05 (*p* < 0.05). A trend was noted at a probability lower than 0.10 but higher than 0.05. To assess the within-subject correlations between urinary metabolites and sleep quality and mood measures, a univariate model with sleep and mood measures as the dependent variable, urinary metabolites as the covariate, and subject as the fixed factor were performed (35). Correlations with *p* < 0.05 and *R*-value > ± 0.4 were considered significant (36). All data are presented as means ± standard errors of the mean (SEM).

## 3. Results

### 3.1. Participants

Twenty-four young men (18–35 years) completed the study: 12 with good sleep quality (age: mean ± SEM: 29 ± 0.9 years; body weight: 79.3 ± 2.8 kg; BMI: 24.4 ± 0.6 kg/m^2^) and 12 with poor sleep quality (29 ± 1.2 years; 76.4 ± 2.2 kg; 24.3 ± 0.7 kg/m^2^). The demographics between the groups were similar except for PSQI which was significantly lower in good sleepers (3 ± 1) compared to poor sleepers (8 ± 2, *p* = 0.005) (Table 2). Main effects of sleeper type were identified for evening sleepiness (SSS) [*F*(2, 22) = 5.71 *p* = 0.026], getting to sleep (LSEQ) [*F*(2, 22) = 4.57 *p* = 0.044] and quality of sleep (LSEQ) [*F*(2, 22) = 7.91 *p* = 0.010]. Regardless of treatment, good sleepers rated themselves sleepier in evening (mean ± SEM across treatments; 4.4 ± 0.3) compared to poor sleepers (3.4 ± 0.3; *post hoc p* = 0.026). Good sleepers rated themselves as harder getting to sleep and had worse sleep quality (mean ± SEM across treatments; 47.8 ± 2.6, 46.6 ± 2.7 respectively) compared to poor sleepers (55.6 ± 2.6; *post hoc p* = 0.044, 57.2 ± 2.7; *post hoc p* = 0.010). All subjects completed and tolerated the study protocol. All participants had not been diagnosed with COVID-19.

**Table 2:** Characteristics of participants. Data presented as mean ± SEM, range, median (IQR).

Characteristics	Whole group (*n* = 24)	Poor sleeper (*n* = 12)	Good sleeper (*n* = 12)
*Age, y*	29 ± 4, 19–34, 28.5 (2)	29 ± 3, 19–34, 28 (5.3)	29 ± 3, 24–34, 28.5 (2)
*Body weight, kg*			77.8 ± 8.7, 77.2 (12.2)	76.4 ± 7.7, 76.6 (6.3)	79.3 ± 9.8, 79.8 (14.8)
*BMI, kg/m^2^*	24.3 ± 2.3, 24.1 (2.9)	24.3 ± 2.5, 24.2 (2.3)	24.4 ± 2.2, 24.1 (2.9)
*PSQI Score*	5 ± 3, 1–12, 3 (1.3)	8 ± 2, 6–12, 7 (2.5)***	3 ± 1, 1–5, 3 (1.3)
*Daily screen time, hours*	8.0 ± 3.6, 2–14, 6 (5.1)	9.1 ± 3.9, 2.5–14, 10 (5.75)	8.0 ± 3.6, 3–12.5, 6 (5.13)
*Daily caffeine intake, cups*	1.7 ± 1.5, 0–5, 1.0 (1.75)	2.0 ± 1.6, 0–5, 1.5 (2.13)	1.4 ± 1.3, 0–4, 1.0 (1.63)
*Ethnicity (n)*			
European	15	8	7
Asian	3	0	3
Indian	3	1	2
African	1	1	0
Latin American	2	2	0

BMI, Body Mass Index; PSQI, Pittsburgh Sleep Quality Index (poor sleeper was defined as a PSQI score of >5). ^***^significant difference between poor and good sleepers, *p* < 0.005. SEM, standard error of mean.

### 3.2. Subjective measures of sleep and mood

#### 3.2.1. Sleep measures

**Figure 3:**
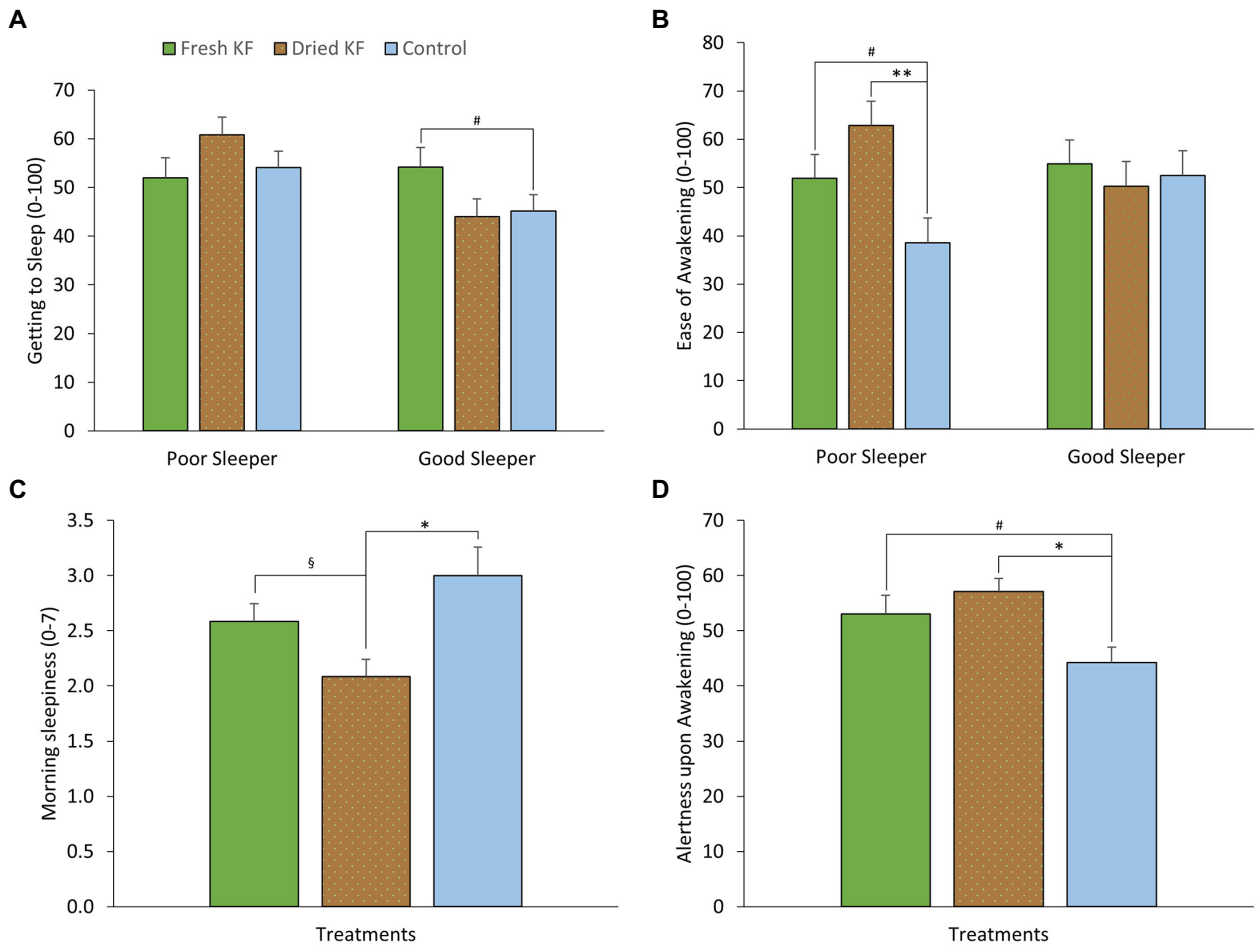
Estimated marginal means and standard error of means (SEM) for post-treatment subjective ratings of morning sleepiness (SSS) **(A)**, getting to sleep (LSEQ) **(B)**, ease of awakening (LSEQ) **(C)** and alertness upon awakening (LSEQ) **(D)**. ^*^p < 0.05 represents statistical difference from control values. ^§^*p* < 0.05 represents the statistical difference from Dried KF values. ^**^p < 0.01 represents statistical difference from control values. ^#^*p* < 0.1 represents statistical difference from control values.

Table 3 shows a summary of subjective sleep measures. Analyzes determined interaction effects of treatment by sleeper type for ease of getting to sleep (LSEQ) [*F*(2, 22) = 4.81, *p* = 0.018] and ease of awakening following sleep (LSEQ) [*F*(2, 22) = 3.85, *p* = 0.037]. Within good sleepers, ratings of ease of getting to sleep tended to increase after the fresh KF treatment (54.1 ± 4.1) compared to the control (45.1 ± 3.4; *post hoc p* = 0.053; Figure 3A). Within poor sleepers, ratings of ease of waking were higher after the freeze-dried KF (62.8 ± 5.1; *post hoc p* = 0.005) and tended to be higher after the fresh KF treatment (51.9 ± 5.0, *post hoc p* = 0.052), compared to the control (38.6 ± 5.1, Figure 3B).

**Table 3:** Subjective sleep outcomes for the fresh KF, dried KF and water control treatments in poor and good sleepers.

Variable	Treatment	Poor sleeper (*n* = 12)		Good sleeper (*n* = 12)		Main effects		
		Mean	SEM	Mean	SEM		*F*	*p*
Evening sleepiness (1–7)^1^	Fresh KF	3.5	0.3	4.8	0.3	Treatment	0.51	0.608
	Dried KF	3.3	0.4	4.3	0.4	Sleeper type	5.70	0.026
	Control	3.5	0.4	4.2	0.4	Treatment × Sleeper type	0.36	0.699
Morning sleepiness (1–7)^1^	Fresh KF	2.6	0.2	2.6	0.2	Treatment	5.47	0.012
	Dried KF	1.8	0.2	2.4	0.2	Sleeper type	0.15	0.706
	Control	3.2	0.4	2.8	0.4	Treatment × Sleeper type	1.94	0.167
Getting to sleep (0–100)^2^	Fresh KF	52.0	4.1	54.1	4.1	Treatment	1.00	0.383
	Dried KF	60.8	3.6	44.0	3.6	Sleeper type	4.57	0.044
	Control	54.1	3.4	45.1	3.4	Treatment × Sleeper type	4.81	0.018
Quality of sleep (0–100)^2^	Fresh KF	60.0	5.3	53.1	5.3	Treatment	1.48	0.249
	Dried KF	58.0	3.9	41.9	3.9	Sleeper type	7.91	0.010
	Control	53.6	4.4	44.7	4.4	Treatment × Sleeper type	0.54	0.589
Ease of awakening (0–100)^2^	Fresh KF	51.9	5.0	54.9	5.0	Treatment	3.23	0.059
	Dried KF	62.8	5.1	50.3	5.1	Sleeper type	0.07	0.787
	Control	38.6	5.1	52.5	5.1	Treatment × Sleeper type	3.85	0.037
Alertness upon awakening (0–100)^2^	Fresh KF	55.7	4.8	50.5	4.8	Treatment	5.11	0.015
	Dried KF	63.9	3.4	50.2	3.4	Sleeper type	2.28	0.145
	Control	42.8	4.0	45.7	4.0	Treatment × Sleeper type	1.96	0.165

1 Data obtained from SSS, Higher score indicates increased feeling of sleepiness.

2 Data obtained from LSEQ, Higher scores indicate a better sleep quality domain.

*Post hoc* estimated marginal means and standard error of means (SEM) are presented with *F* and *p* values of the main effects and interaction from the linear mixed models.

Main effects of treatment were identified for morning sleepiness (SSS) [*F* (2, 22) = 5.47, *p* = 0.012] and alertness upon awakening (LSEQ) [*F*(2, 22) = 5.11, *p* = 0.015]. Irrespective of sleeper type, morning sleepiness ratings were higher after the control (3.00 ± 0.26; *post hoc p* = 0.013) and fresh KF (2.6 ± 0.2; *post hoc p* = 0.034) interventions compared to the freeze-dried KF treatment (2.1 ± 0.2; Figure 3C). Ratings of alertness upon awakening were higher after the freeze-dried KF (57.1 ± 2.4; *post hoc p* = 0.018) and tended to be higher following the fresh KF treatment (53.1 ± 3.4; *post hoc p* = 0.082) compared to the control (44.2 ± 2.8, Figure 3D).

#### 3.2.2. Mood measures

**Figure 4:**
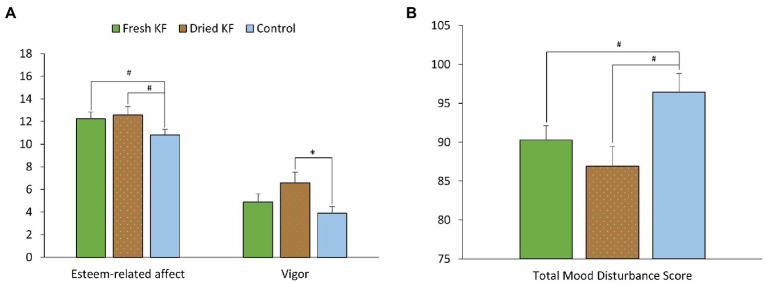
Estimated marginal means and standard error of means (SEM) for post-treatment subjective mood ratings (abbreviated Profile of Mood States) of esteem and vigor **(A)** and total mood disturbance **(B)**. ^*^p < 0.05 represents statistical difference from control values. ^#^*p* < 0.1 represents statistical difference from control values.

A summary of subjective mood measures is presented in Table 4. Main significant effects of treatment for esteem-related affect were identified [*F*(2, 22) = 4.56, *p* = 0.022], vigor [*F*(2, 22) = 4.34, *p* = 0.026], and total mood disturbance [*F*(2, 22) = 3.82, *p* = 0.038]. Furthermore, main trend effects of treatment for fatigue [*F*(2, 22) = 3.17, *p* = 0.062] and confusion [*F*(2, 22) = 3.37, *p* = 0.053] were identified. Irrespective of sleeper type, ratings for esteem-related affect tended to be higher following freeze-dried KF (12.6 ± 0.8; *p* = 0.060) and fresh KF (12.3 ± 0.6; *post hoc p* = 0.054) compared to the control (10.8 ± 0.5; Figure 4A). Ratings for vigor were higher following freeze-dried KF (6.58 ± 0.91; *post hoc p* = 0.030), but not fresh KF (4.9 ± 0.7; *post hoc p* = 0.487) treatments compared to the control (3.9 ± 0.6; Figure 4A). Ratings for total mood disturbance tended to be lower following the freeze-dried KF (86.9 ± 2.5; *post hoc p* = 0.054) and fresh KF (90.3 ± 1.8; *post hoc p* = 0.063) treatments compared to the control (96.4 ± 2.1, Figure 4B). No main effects for sleeper type or interactions were identified for any mood measures.

**Table 4:** Subjective mood outcomes for the fresh KF, dried KF and water control treatments in poor and good sleepers.

Variable	Treatment	Poor sleeper (*n* = 12)		Good sleeper (*n* = 12)		Main effects		
		Mean	SEM	Mean	SEM		*F*	*p*
Tension (0–24)	Fresh KF	2.4	0.7	1.8	0.7	Treatment	1.09	0.352
	Dried KF	2.3	0.8	2.3	0.8	Sleeper type	0.51	0.482
	Control	2.9	0.6	1.8	0.6	Treatment × Sleeper type	1.10	0.350
Anger (0–24)	Fresh KF	0.8	0.4	0.8	0.4	Treatment	1.85	0.180
	Dried KF	0.5	0.3	0.5	0.3	Sleeper type	0.04	0.850
	Control	1.7	0.8	1.4	0.8	Treatment × Sleeper type	0.02	0.976
Fatigue (0–20)	Fresh KF	2.5	0.7	1.9	0.7	Treatment	3.17	0.062
	Dried KF	2.1	0.5	2.1	0.5	Sleeper type	1.58	0.222
	Control	4.6	0.9	2.8	0.9	Treatment × Sleeper type	0.70	0.508
Depression (0–28)	Fresh KF	0.7	0.6	0.9	0.5	Treatment	1.77	0.193
	Dried KF	0.6	0.6	0.8	0.6	Sleeper type	0.01	0.909
	Control	2.0	0.9	1.2	0.8	Treatment × Sleeper type	0.36	0.699
Esteem-related affect (0–24)	Fresh KF	11.9	0.8	12.6	0.8	Treatment	4.56	0.022
	Dried KF	12.6	1.1	12.6	1.1	Sleeper type	0.41	0.530
	Control	10.3	0.7	11.4	0.7	Treatment × Sleeper type	0.36	0.703
Vigor (0–20)	Fresh KF	4.9	1.0	4.8	1.0	Treatment	4.34	0.026
	Dried KF	6.6	1.3	6.6	1.3	Sleeper type	0.01	0.921
	Control	3.7	0.9	4.1	0.9	Treatment × Sleeper type	0.08	0.928
Confusion (0–20)	Fresh KF	1.8	0.7	2.0	0.7	Treatment	3.37	0.053
	Dried KF	0.8	0.3	0.8	0.3	Sleeper type	0.03	0.869
	Control	2.5	0.8	1.9	0.8	Treatment × Sleeper type	0.60	0.560
Total mood disturbance	Fresh KF	91.0	2.6	89.6	2.6	Treatment	3.82	0.038
	Dried KF	86.9	3.6	86.9	3.6	Sleeper type	0.68	0.417
	Control	99.3	3.4	93.5	3.4	Treatment × Sleeper type	0.43	0.654

*Post hoc* estimated marginal means and standard error of means (SEM) are presented with *F* and *p* values of the main effects and interaction from the linear mixed models.

### 3.3. Objective measures of sleep quality

**Figure 5:**
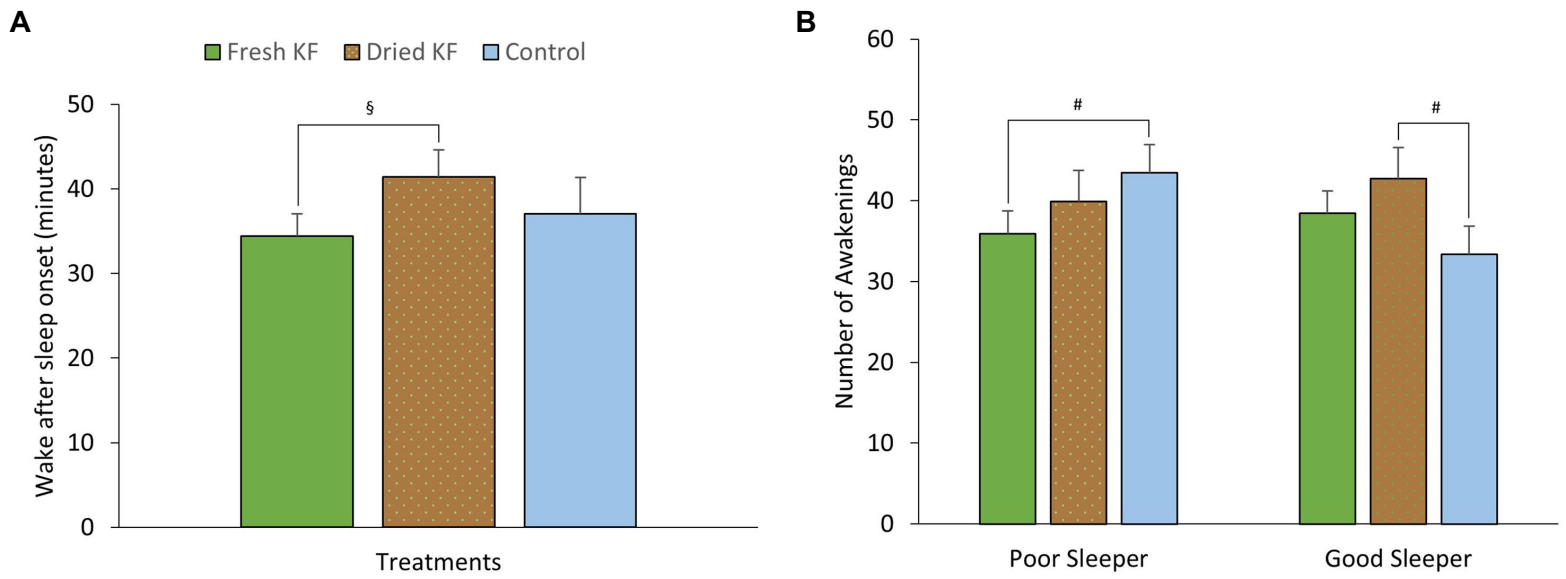
Estimated marginal means and standard error of means (SEM) for post-treatment objective sleep (actigraphy) measures of wake after sleep onset **(A)** and the number of awakening **(B)**. ^§^*p* < 0.05 represents the statistical difference from Dried KF values. ^#^*p* < 0.1 represents statistical difference from control values.

A summary of objective sleep measures is presented in Table 5. There was an interaction effect between treatment and sleeper type for the number of awakenings [*F*(2, 22) = 4.06, *p* = 0.032]. Within poor sleepers, the number of awakenings tended to be lower after the fresh KF treatment (35.9 ± 2.76; *p* = 0.099) than the control (43.4 ± 3.5, Figure 5B). Conversely, among good sleepers, the number of awakenings tended to increase after the freeze-dried KF treatment (42.8 ± 3.81; *post hoc p* = 0.080) compared to the control (33.3 ± 3.54, Figure 5B).

**Table 5:** Objective sleep outcomes for the fresh KF, dried KF and water control treatments in poor and good sleepers.

Variable	Treatment	Poor sleeper (*n* = 12)		Good sleeper (*n* = 12)		Main effects		
		Mean	SEM	Mean	SEM		*F*	*p*
Latency (minutes)	Fresh KF	17.3	4.3	8.8	4.3	Treatment	1.45	0.256
	Dried KF	10.6	3.4	12.1	3.4	Sleeper type	0.49	0.491
	Control	20.8	6.5	17.0	6.5	Treatment × Sleeper type	1.23	0.312
Efficiency (%)	Fresh KF	85.4	1.6	87.3	1.6	Treatment	0.31	0.740
	Dried KF	86.4	1.2	85.7	1.2	Sleeper type	0.15	0.698
	Control	84.9	1.9	85.6	1.9	Treatment × Sleeper type	0.46	0.638
Total sleep time (minutes)	Fresh KF	389.3	15.3	400.1	15.3	Treatment	0.67	0.520
	Dried KF	409.5	13.3	403.2	13.3	Sleeper type	0.01	0.938
	Control	406.8	20.9	398.1	20.9	Treatment × Sleeper type	0.32	0.731
Wake after sleep onset (minutes)	Fresh KF	35.4	3.8	33.4	3.8	Treatment	3.74	0.040
	Dried KF	41.1	4.5	41.7	4.5	Sleeper type	0.64	0.433
	Control	43.1	6.1	31.0	6.1	Treatment × Sleeper type	1.37	0.274
Number of awakenings	Fresh KF	35.9	2.8	38.4	2.8	Treatment	1.52	0.240
	Dried KF	39.9	3.8	42.8	3.8	Sleeper type	0.17	0.684
	Control	43.4	3.5	33.3	3.5	Treatment × Sleeper type	4.06	0.032
Average awakening length (minutes)	Fresh KF	1.0	0.1	0.9	0.1	Treatment	1.43	0.260
	Dried KF	1.0	0.1	1.0	0.1	Sleeper type	0.36	0.553
	Control	1.0	0.1	0.9	0.1	Treatment × Sleeper type	0.17	0.845

*Post hoc* estimated marginal means and standard error of means (SEM) are presented with *F* and *p* values of the main effects and interaction from the linear mixed models.

There were main effects of treatment for wake after sleep onset [*F*(2, 22) = 3.74, *p* = 0.040]. Irrespective of sleeper type, the number of awakenings was higher after the freeze-dried KF treatment (41.4 ± 3.2; *p* = 0.045) but not the fresh KF treatment (34.4 ± 2.7; *p* = 0.888) compared to the control (37.0 ± 4.3) (Figure 5A). No main effects of treatment for sleeper type were identified for any objective sleep measures.

### 3.4. Urinary metabolites

**Figure 6:**
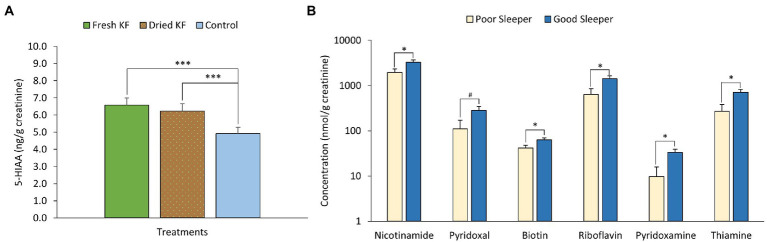
Estimated marginal means and standard error of means (SEM) for post-treatment urinary 5-HIAA **(A)** and urinary B-vitamins (B). ^#^*p* < 0.1 represents statistical difference between sleeper type. ^*^p < 0.05 represents the statistical difference between sleeper types. ^***^p < 0.005 represents statistical difference from control concentrations.

A summary of urinary measures is in Table 6. A significant main effect of treatment for urinary 5-HIAA concentration was identified [*F*(2, 22) = 19.15, *p* < 0.001] with *post hoc* comparisons identifying freeze-dried KF (6.2 ± 0.4 mg/g creatinine; *p* < 0.001) and fresh KF (6.6 ± 0.4 mg/g creatinine; *p* = 0.001) treatments as showing significantly higher 5-HIAA concentration, as compared to the control (4.9 ± 0.4 mg/g creatinine) (Figure 6A). A significant main effect of sleeper type for urinary B-vitamin concentration was identified for nicotinamide [*F*(2, 22) = 6.11, *p* = 0.022], biotin [*F*(2, 22) = 5.77, *p* = 0.025], riboflavin [*F*(2, 22) = 6.85, *p* = 0.016], pyridoxamine [*F*(2, 22) = 7.71, *p* = 0.011], thiamine [*F*(2, 22) = 7.58, *p* = 0.012] and was near significant for pyridoxal [*F*(2, 22) = 4.28, *p* = 0.051]. The *post hoc* comparison revealed that good sleepers had significantly higher concentrations of all measured B-vitamins than poor sleepers (Figure 6B). No interaction effects were identified for any urinary metabolite measures. Additionally, the urinary concentration of 5-HIAA and vitamin C in good sleepers was negatively related to total mood disturbance (5-HIAA; *r* = −0.41, *p* = 0.04) and latency (vitamin C; *r* = −0.72, *p* < 0.005). A full within correlation table is presented in Supplementary Table S1.

**Table 6:** Urinary 5-HIAA, aMT6s, vitamin C and B-vitamins for the fresh KF, dried KF and water control treatments in poor and good sleepers.

Variable	Treatment	Poor sleeper (*n* = 12)		Good sleeper (*n* = 12)		Main effects		
		Mean	SEM	Mean	SEM		*F*	*p*
5-HIAA (ng/g creatinine)	Fresh KF	6.6	0.6	6.5	0.6	Treatment	19.15	0.001
	Dried KF	5.7	0.6	6.7	0.6	Sleeper type	0.30	0.591
	Control	4.8	0.5	5.0	0.5	Treatment × Sleeper type	1.72	0.202
6aMTs (ng/g creatinine)	Fresh KF	49570.4	5771.4	39643.6	5771.4	Treatment	0.27	0.769
	Dried KF	45419.6	4522.9	40818.7	4522.9	Sleeper type	1.02	0.323
	Control	46983.1	5035.9	40856.7	5035.9	Treatment × Sleeper type	0.79	0.466
Vitamin C (μmol/mmol creatinine)	Fresh KF	26.5	15.0	27.9	15.0	Treatment	1.98	0.161
	Dried KF	24.0	17.6	27.3	17.6	Sleeper type	0.00	0.983
	Control	13.9	7.2	8.1	7.2	Treatment × Sleeper type	0.06	0.938
Pantothenic acid (nmol/g creatinine)	Fresh KF	5487.8	1174.3	7107.5	1174.3	Treatment	0.03	0.975
	Dried KF	5753.4	1185.6	6449.9	1185.6	Sleeper type	0.92	0.349
	Control	5511.2	1173.2	6597.3	1173.2	Treatment × Sleeper type	0.12	0.887
4-pyridoxic acid (nmol/g creatinine)	Fresh KF	4240.8	1074.6	5558.6	1074.6	Treatment	1.73	0.201
	Dried KF	3975.4	478.3	3455.3	478.3	Sleeper type	0.28	0.604
	Control	4130.5	1016.3	4753.7	1016.3	Treatment × Sleeper type	1.05	0.367
Nicotinamide (nmol/g creatinine)	Fresh KF	1889.1	879.5	3959.6	879.5	Treatment	0.55	0.587
	Dried KF	1962.6	455.4	2871.0	455.4	Sleeper type	6.11	0.022
	Control	1973.3	409.3	3056.1	409.3	Treatment × Sleeper type	0.68	0.515
Pyridoxal (nmol/g creatinine)	Fresh KF	121.9	47.9	273.9	47.9	Treatment	0.62	0.549
	Dried KF	109.7	58.0	260.0	58.0	Sleeper type	4.28	0.051
	Control	99.4	79.5	316.4	79.5	Treatment × Sleeper type	1.06	0.363
Biotin (nmol/g creatinine)	Fresh KF	42.5	10.4	67.0	10.4	Treatment	0.37	0.694
	Dried KF	41.7	9.3	57.3	9.3	Sleeper type	5.77	0.025
	Control	40.8	9.9	66.1	9.9	Treatment × Sleeper type	0.38	0.688
Riboflavin (nmol/g creatinine)	Fresh KF	505.2	466.2	2112.3	466.2	Treatment	1.43	0.261
	Dried KF	600.4	230.4	938.7	230.4	Sleeper type	6.85	0.016
	Control	820.9	328.3	1226.8	328.3	Treatment × Sleeper type	1.72	0.202
Pyridoxamine (nmol/g creatinine)	Fresh KF	9.9	7.0	35.9	7.0	Treatment	0.71	0.504
	Dried KF	9.4	7.9	34.8	7.9	Sleeper type	7.71	0.011
	Control	10.2	4.7	28.9	4.7	Treatment × Sleeper type	0.54	0.593
Thiamine (nmol/g creatinine)	Fresh KF	265.6	181.4	923.1	181.4	Treatment	0.92	0.414
	Dried KF	298.6	155.4	547.5	155.4	Sleeper type	7.58	0.012
	Control	242.0	222.6	672.1	222.6	Treatment × Sleeper type	1.48	0.250

*Post hoc* estimated marginal means and standard error of means (SEM) are presented with *F* and *p* values of the main effects and interaction from the linear mixed models.

## 4. Discussion

The current study shows that irrespective of the sleep quality participant experience, acutely supplemented freeze-dried KF resulted in feeling less sleepy and more alert. Both fresh and freeze-dried KF increased urinary excretion of 5-HIAA. Furthermore, poor sleepers felt it was easier to wake up in the morning after consuming freeze-dried KF in the evening and this was also moderately felt when consuming the fresh KF. Good sleepers rated themselves as getting to sleep easier when consuming the fresh KF. Interestingly, poor sleepers tended to wake more often in the night after consuming freeze-dried KF with the evening meal, while good sleepers woke less often during the night after consuming fresh KF with the evening meal.

The beneficial impacts observed when consuming KF on aspects of sleep quality are consistent with other KF sleep intervention studies that showed improvement in subjective daytime function ratings (37) and sleep quality measures (21). In addition, in a mouse model fed KF skin extracts sleep onset latency was reduced (38), thus providing further evidence supporting the beneficial impact of KF on sleep. Sleep is a complex process controlled by internal and external factors. There are potentially at least three explanations that could explain the observed outcomes. Firstly, it is known that KF is rich in phenylalanine, tyrosine, tryptophan, glutamic acid (22) and serotonin (39) that are further metabolized to produce dopaminergic, serotoninergic, and GABAergic neurochemicals. These neurotransmitters are vital in the regulation of sleep/wake cycles. Secondly, green KF contains the enzyme actinidin, which has been shown to cause an earlier peak increase in plasma circulating amino acids in adults when compared to kiwifruit without actinidin (40). Therefore, potentially increasing the concentration of bioavailable amino acids to be further metabolized into neurochemicals. Lastly, KF may act on improving sleep *via* potentiating the bioaminergic responses. This has been demonstrated in *in vitro* studies (41). Given this, KF may impact sleep through the supply and modulation of peripheral neurochemical responses, which may impact systemic concentration modifications throughout the rest of the body. Further studies are needed to determine which postprandial biomarkers are increased in sleep and upon waking the following morning to elucidate how KF may impact sleep and subsequent mood.

The current study also shows that irrespective of sleep quality participant experience, acutely supplemented freeze-dried KF resulted in improved vigor compared to the control. Likewise, fresh, and freeze-dried KF treatments tended to improve esteem-related affects, and freeze-dried KF tended to decrease total mood disturbance score compared to the control. The impacts of consuming KF on aspects of waking mood in this study are consistent with earlier KF mood studies that showed improvement in mood (42, 43).

Similarly, the various underlying regulatory mechanisms of mood are complex. Firstly, vitamin C is a potent water-soluble antioxidant with distinct roles in the body, including reducing systemic inflammation and as co-factors in production of neurotransmitters. Consumption of KF reduces oxidative stress and inflammatory markers (44). Additionally, higher vitamin C status has been correlated with decreased total mood disturbances (45), and regular consumption of KF increases vitamin C in the body (46). Secondly, neurochemicals, such as serotonin, provided by KF, may also enhance mood (47). Lastly, KF is rich in other vitamins and polyphenols, which may have facilitated these mood improvements by improving oxidative stress and metabolism. Given this, KF may have impacted mood *via* the supply of vitamin C and other nutrients, which altered the metabolism of neurochemicals and may also have affected oxidative stress. One cannot rule out that improved sleep might have led to improved mood outcomes and vice versa (48).

No differences between KF treatments were observed for urinary aMT6s and vitamin C. The correlation analysis of urinary metabolites with sleep and mood measures indicated that urinary aMT6s and vitamin C morning excretion were negatively related to sleep latency. Waking urinary aMT6s can be used to predict nocturnal plasma melatonin (13) and lower urinary aMT6s excretion has been associated with lowered alertness (11). The results of aMT6s can be interpreted in two ways; first, the lack of treatment effects on excreted aMT6s may be due to insufficient samples collected or only one sample collected upon waking. Studies have shown that a 24–48-h collection period may better reflect these changes in urinary aMT6s (49). Dim light melatonin onset, a circadian phase marker measured over a 5–7 h window in plasma or saliva before bed, could be measured to assess whether KF may alter the circadian phase. Second, the finding may suggest that another mechanism not related to melatonin may facilitate the acute improvements in sleep quality and mood by KF. However, regardless of sleep quality, consumption of fresh and dried KF treatments increased urinary excretion of 5-HIAA compared to the control, which is consistent with data on the acute effects of KF consumption on urinary 5-HIAA (20). Comparably, in a different study, participants consuming tart cherries increased morning urinary 5-HIAA excretion and improved mood (19). 5-HIAA is the primary metabolite of serotonin and is involved with mood regulation (47). The results here suggested that consumption of fresh or freeze-dried KF may have increased circulating and brain levels of serotonin, which impacted mood.

Concentrations of excreted B-vitamins were greater for good sleepers than for poor sleepers, regardless of the treatment received. Excretion of B-Vitamins can be used as an indicator of baseline B-vitamin status, with higher excretion suggesting saturation and lowered amounts indicating deficiency (50). The lower amount of B-vitamins may be explained by a lower background dietary intake of B-vitamins, however this is speculation and should be interpreted with caution as no dietary records were collected. It may be suggested that poor sleepers utilize more B-vitamins for cellular metabolic processes. For example, nicotinamide (a form of B_3_), pyridoxine and pyridoxamine (forms of B_6_) are required as co-factors in metabolizing neurotransmitters (51). Additionally, low levels of B-vitamins have also been associated with low-grade inflammation (52) and poor sleep caused increased circulating inflammatory markers (53). However, this study did not measure any inflammatory markers, suggesting that inflammatory status is worth exploring in future studies. Other factors that were not assessed that may also impact B-vitamins include exercise status, which is known to increase the requirements for B-vitamins (54).

Irrespective of sleep quality participant experience, acutely supplemented freeze-dried KF improved morning sleepiness compared to both fresh KF treatment and control. This finding could be explained by the structural and compositional differences between KF interventions; a KF drink that contained the skin (liquid) or KF flesh eaten fresh without the skin (solid). Including skin in the freeze-dried powder altered the polyphenolic content, and as mentioned previously, KF skin extracts potentiate sleep induction in a pentobarbital-induced sleep mouse model (38). Thus, the freeze-dried powder may be improving sleep onset, thus causing better morning alertness. The results on sleep onset suggested that this may occur in poor sleepers. Furthermore, the added polyphenols may be impacting the body by affecting the expression of clock genes (55), improving cerebral blood flow (thus, improving mood (56)), altering the permeability of the blood–brain barrier and/or altering neurotransmissions (57).

Nevertheless, it is worth noting that the food structure may influence stomach emptying rate and satiety and cause differential interactions with other food matrices, consequently impacting gut comfort. For example, consumption of a carbohydrate-rich meal with KF lowered postprandial hunger compared to consumption of a carbohydrate meal only (58). Also, consuming fresh green actinidin-containing KF has improved gastric comfort compared to KF without actinidin (59). These findings suggest that improvements in sleep quality may be due to reduced feelings of bloating and hunger; however, these parameters were not measured in this study.

Contrary to the previous KF and sleep quality studies, this study was the first to assess the acute and separate effects of a fresh KF or dried (containing skin) KF treatment on sleep quality and mood in a healthy male cohort with good or poor-quality sleep. The urinary 5-HIAA and aMT6s concentrations suggest a novel potential mechanism underpinning the relationship between KF and sleep. Additionally, the results provide evidence for the repurposing of KF skin and lower-quality fruit, which would otherwise be wasted, into products that can easily be stored with extended shelf-life.

This study is not without limitations. One of the limitations was the low subject numbers, the study was powered using a different intervention to this (34), as this was the first study to assess acute impact of KF on LSEQ. Secondly, due to the nature of the intervention, it was impossible to blind participants to their treatment. This is evident in the ratings for getting to sleep and quality of sleep in good sleepers. Fresh KF was on average higher than both the control and freeze-dried KF, suggesting that some participants may have preconceived notions about one treatment over another. Thirdly, because the study was conducted only on men, it is difficult to interpret the results for women. Fourthly, the control used was not iso-calorically matched, suggesting participants may have rated themselves poorly on the control intervention due to other factors such as appetite and hunger, which were not measured. Furthermore, urine samples were limited to morning samples, with no other timepoints collected. Collecting samples other than urine at different time points may have allowed a better understanding of the postprandial changes that have occurred. Collection of plasma and saliva pre and post intervention and up to 5 h before bedtime may have provided a better understanding of melatonin circulation, which may have influenced sleep (49). In addition, saliva could have been collected upon waking and used to quantify cortisol awakening response (CAR), which could provide a simple measure of the reactive capacity of the hypothalamic–pituitary–adrenal (HPA) axis.

Although actigraphy was used here and in other acute settings (60), results should be interpreted with caution. For instance, a person lying still while awake may be recorded as asleep due to immobility. Actigraphy does not account for these events. Using polysomnography was not feasible in this study due to the in-home setting, but would have provided a better understanding of the actual acute impacts of KF and sleep quality and architecture (61). Future studies could incorporate a larger cohort with participants of mixed cohort of males and females consuming differing doses of the freeze-dried KF. Furthermore, future studies could use an energy matched placebo with the same amounts of vitamin C and B-vitamins. Additionally, other cohorts worth exploring include older adults due to age-related sleep disturbances (62), University students under high stress (63), or inpatients in planned care services (64).

## 5. Conclusion

Overall, this study is the first to demonstrate that a single evening meal with KF, whether fresh or freeze-dried, improved sleep quality and mood in males with good or poor-quality sleep. This effect may have been mediated through increased serotonin metabolism. Further studies should be conducted to elucidate the impact of freeze-dried KF on those who experience poor sleep and the potential mechanisms by measuring other biomarkers in urine, plasma, and saliva. Nonetheless, as beneficial effects of sleep were identified following supplementation with KF in young men, these data help provide additional evidence for the role of KF in facilitating healthy sleep regulation.

## Data availability statement

The original contributions presented in the study are included in the article/Supplementary material, further inquiries can be directed to the corresponding author.

## Ethics statement

The study protocol was approved by the Massey University Human Ethics Committee study (Massey University HEC: Southern A application-20/52), and the trial has been registered at the Australian New Zealand Clinical Trials Registry (ANZCTR) (ANZCTR12620000411943). Participants provided written, informed consent for participation.

## Author contributions

AK, CG, NR, WM, and SH conceptualized the study and contributed to editing the manuscript. AK designed and conducted the study and prepared the manuscript with input from the other authors. All authors contributed to the article and approved the submitted version.

## Funding

This study was funded by the Riddet Institute, a Centre of Research Excellence (CoRE) funded by the NZ Tertiary Education Commission, and the High-Value Nutrition National Science Challenge (UOAX1902) with in-kind support from the partner organization: Zespri International and Alpha Group Holdings. The funders had no role in the design of this study and did not have any role during its execution, analyses, interpretation of the data, or decision to submit results. AK was supported by a PhD stipend from the Riddet Institute.

## Conflict of interest

The authors declare that the research was conducted in the absence of any commercial or financial relationships that could be construed as a potential conflict of interest.

## Publisher’s note

All claims expressed in this article are solely those of the authors and do not necessarily represent those of their affiliated organizations, or those of the publisher, the editors and the reviewers. Any product that may be evaluated in this article, or claim that may be made by its manufacturer, is not guaranteed or endorsed by the publisher.

## Footnotes

^1^www.anzctr.org.au, ACTRN12621000046808.

## Abbreviations

KF: kiwifruit
5-HIAA: 5-hydroxyindoleacetic acid
aMT6s: 6-sulfatoxymelatonin
LSEQ: Leeds Sleep Evaluation Questionnaire
SSS: Stanford Sleepiness Scale
BMI: Body Mass Index
PSQI: Pittsburgh Sleep Quality Index
POMS: Profile of Mood States
VAS: Visual Analogue Scales
TMD: Total Mood Score
HPLC: high-performance liquid chromatography
CAR: cortisol awakening response
HPA: hypothalamic–pituitary–adrenal
