# Supplementary material for: Acute effects of fresh versus dried Hayward green kiwifruit on sleep quality, mood, and sleep-related urinary metabolites in healthy young men with good and poor sleep quality
Source: Front Nutr. 2023 Mar 14;10:1079609. doi: 10.3389/fnut.2023.1079609 (PMC10043399; doi:10.3389/fnut.2023.1079609)
Supplement: Supplementary file 1 [file Table_1.DOCX]

Supplementary Material

# Supplementary Data

## Supplementary Table

## Table S1 Within-subject correlations between urinary 5-HIAA, aMT6s and Vitamin C and subjective and objective measures of sleep quality and subjective mood in poor and good sleepers.

|  |  | Within-subjects correlations | | | | | |
| --- | --- | --- | --- | --- | --- | --- | --- |
|  |  | Combined | | Poor sleeper  (n= 12) | | Good sleeper (n= 12) | |
|  |  | *r* | *P* | *r* | *P* | *r* | *P* |
| **5-HIAA** | |  |  |  |  |  |  |
| *Subjective Sleep Quality* | |  |  |  |  |  |  |
|  | Evening Sleepiness | -0.20 | 0.17 | -0.34 | 0.09 | -0.10 | 0.64 |
|  | Morning Sleepiness | -0.12 | 0.12 | -0.14 | 0.50 | -0.09 | 0.66 |
|  | Getting to Sleep | -0.06 | 0.70 | -0.02 | 0.92 | -0.10 | 0.63 |
|  | Quality of Sleep | 0.03 | 0.85 | 0.09 | 0.68 | -0.03 | 0.87 |
|  | Ease of Awakening | -0.06 | 0.70 | -0.02 | 0.92 | -0.12 | 0.56 |
|  | Alertness Upon Awakening | 0.14 | 0.35 | 0.24 | 0.25 | 0.00 | 0.99 |
| *Objective Sleep Quality* | |  |  |  |  |  |  |
|  | Latency | -0.24 | 0.09 | -0.09 | 0.66 | -0.35 | 0.08 |
|  | Efficiency | 0.11 | 0.47 | -0.04 | 0.85 | 0.22 | 0.30 |
|  | Total Sleep Time | -0.07 | 0.66 | -0.17 | 0.41 | 0.08 | 0.70 |
|  | Wake after sleep onset | -0.07 | 0.64 | -0.36 | 0.08 | 0.29 | 0.15 |
|  | Number of Awakenings | 0.03 | 0.81 | -0.29 | 0.15 | 0.38 | 0.06 |
|  | Average Awakening Length | -0.10 | 0.51 | -0.25 | 0.24 | 0.04 | 0.86 |
| *Subjective Mood* | |  |  |  |  |  |  |
|  | Tension | -0.05 | 0.73 | -0.10 | 0.63 | 0.02 | 0.92 |
|  | Anger | -0.10 | 0.51 | 0.05 | 0.80 | -0.38 | 0.06 |
|  | Fatigue | -0.23 | 0.11 | -0.19 | 0.35 | -0.35 | 0.09 |
|  | Depression | -0.13 | 0.37 | -0.04 | 0.86 | -0.30 | 0.14 |
|  | Esteem-related Affect | 0.21 | 0.15 | 0.24 | 0.25 | 0.19 | 0.37 |
|  | Vigour | -0.18 | 0.21 | 0.22 | 0.29 | 0.21 | 0.30 |
|  | Confusion | -0.08 | 0.58 | 0.03 | 0.89 | -0.20 | 0.33 |
|  | Total Mood Disturbance | -0.21 | 0.14 | -0.14 | 0.49 | -0.41 | 0.04 |
| **6aMTs** | |  |  |  |  |  |  |
| *Subjective Sleep Quality* | |  |  |  |  |  |  |
|  | Evening Sleepiness | 0.19 | 0.19 | 0.00 | 0.99 | 0.35 | 0.09 |
|  | Morning Sleepiness | 0.24 | 0.10 | 0.26 | 0.21 | 0.22 | 0.29 |
|  | Getting to Sleep | 0.14 | 0.33 | -0.34 | 0.10 | 0.14 | 0.51 |
|  | Quality of Sleep | 0.02 | 0.88 | 0.05 | 0.82 | -0.01 | 0.97 |
|  | Ease of Awakening | 0.14 | 0.33 | 0.17 | 0.40 | 0.09 | 0.66 |
|  | Alertness Upon Awakening | 0.16 | 0.27 | 0.19 | 0.36 | 0.12 | 0.56 |
| *Objective Sleep Quality* | |  |  |  |  |  |  |
|  | Latency | -0.29 | 0.05 | 0.22 | 0.29 | -0.35 | 0.09 |
|  | Efficiency | -0.12 | 0.41 | -0.05 | 0.83 | 0.19 | 0.36 |
|  | Total Sleep Time | -0.27 | 0.06 | -0.33 | 0.11 | -0.19 | 0.37 |
|  | Wake after sleep onset | -0.05 | 0.75 | 0.09 | 0.66 | 0.02 | 0.93 |
|  | Number of Awakenings | 0.11 | 0.45 | 0.11 | 0.60 | 0.11 | 0.60 |
|  | Average Awakening Length | 0.02 | 0.91 | -0.05 | 0.81 | 0.09 | 0.68 |
| *Subjective Mood* | |  |  |  |  |  |  |
|  | Tension | 0.09 | 0.54 | 0.33 | 0.10 | -0.33 | 0.11 |
|  | Anger | 0.07 | 0.64 | 0.17 | 0.42 | -0.13 | 0.55 |
|  | Fatigue | 0.08 | 0.57 | 0.15 | 0.47 | -0.05 | 0.80 |
|  | Depression | -0.13 | 0.36 | -0.02 | 0.93 | -0.37 | 0.07 |
|  | Esteem-related Affect | 0.00 | 0.98 | -0.08 | 0.72 | 0.08 | 0.69 |
|  | Vigour | -0.18 | 0.21 | 0.36 | 0.08 | 0.04 | 0.84 |
|  | Confusion | 0.07 | 0.61 | 0.09 | 0.65 | 0.05 | 0.82 |
|  | Total Mood Disturbance | 0.09 | 0.56 | 0.21 | 0.30 | 0.20 | 0.35 |
| **Vitamin C** | |  |  |  |  |  |  |
| *Subjective Sleep Quality* | |  |  |  |  |  |  |
|  | Evening Sleepiness | 0.03 | 0.82 | 0.05 | 0.82 | 0.02 | 0.91 |
|  | Morning Sleepiness | -0.04 | 0.80 | -0.16 | 0.45 | 0.23 | 0.26 |
|  | Getting to Sleep | -0.09 | 0.55 | -0.24 | 0.25 | 0.15 | 0.48 |
|  | Quality of Sleep | -0.13 | 0.38 | -0.26 | 0.21 | 0.04 | 0.83 |
|  | Ease of Awakening | -0.08 | 0.60 | 0.07 | 0.75 | -0.37 | 0.07 |
|  | Alertness Upon Awakening | -0.18 | 0.21 | -0.07 | 0.73 | -0.38 | 0.06 |
| *Objective Sleep Quality* | |  |  |  |  |  |  |
|  | Latency | -0.43 | 0.00 | -0.12 | 0.55 | -0.72 | 0.00 |
|  | Efficiency | 0.13 | 0.37 | -0.03 | 0.88 | 0.29 | 0.16 |
|  | Total Sleep Time | 0.05 | 0.71 | -0.01 | 0.95 | 0.17 | 0.41 |
|  | Wake after sleep onset | 0.17 | 0.24 | 0.13 | 0.55 | 0.25 | 0.23 |
|  | Number of Awakenings | 0.11 | 0.45 | -0.02 | 0.93 | 0.28 | 0.18 |
|  | Average Awakening Length | 0.06 | 0.67 | 0.07 | 0.74 | 0.05 | 0.80 |
| *Subjective Mood* | |  |  |  |  |  |  |
|  | Tension | -0.11 | 0.46 | -0.20 | 0.33 | 0.06 | 0.77 |
|  | Anger | 0.03 | 0.85 | 0.08 | 0.72 | -0.08 | 0.71 |
|  | Fatigue | -0.15 | 0.30 | -0.11 | 0.61 | -0.27 | 0.19 |
|  | Depression | 0.05 | 0.75 | 0.09 | 0.68 | -0.04 | 0.87 |
|  | Esteem-related Affect | 0.22 | 0.13 | 0.27 | 0.19 | 0.16 | 0.43 |
|  | Vigour | 0.18 | 0.21 | 0.12 | 0.56 | 0.27 | 0.20 |
|  | Confusion | -0.05 | 0.73 | -0.23 | 0.28 | 0.19 | 0.37 |
|  | Total Mood Disturbance | -0.15 | 0.31 | -0.13 | 0.53 | -0.20 | 0.33 |
